# Neurological Respiratory Failure

**DOI:** 10.3390/diseases6010007

**Published:** 2018-01-10

**Authors:** Mohan Rudrappa, Laxmi Kokatnur, Oleg Chernyshev

**Affiliations:** 1 Department of Pulmonary and Critical Care, Louisiana State University Health Science Center, Shreveport, LA 71115, USA; 2 Department of Neurology, Louisiana State University Health Science Center, Shreveport, LA 71115, USA; drlaxmisk@gmail.com (L.K.); ochern@lsuhsc.edu (O.C.)

**Keywords:** West Nile virus, diaphragmatic palsy, respiratory failure

## Abstract

West Nile virus infection in humans is mostly asymptomatic. Less than 1% of neuro-invasive cases show a fatality rate of around 10%. Acute flaccid paralysis of respiratory muscles leading to respiratory failure is the most common cause of death. Although the peripheral nervous system can be involved, isolated phrenic nerve palsy leading to respiratory failure is rare and described in only two cases in the English literature. We present another case of neurological respiratory failure due to West Nile virus-induced phrenic nerve palsy. Our case reiterates the rare, but lethal, consequences of West Nile virus infection, and the increase of its awareness among physicians.

## 1. Introduction

The incidence of West Nile virus (WNV) infection is increasing in the United States from its first description three decades ago [1]. Although most cases are symptomatic, around 1% of patients develop West Nile neuro-invasive disease (WNND) with significant mortality and morbidity [2]. Respiratory failure due to acute flaccid paralysis (AFP) caused by WNV strongly correlates with mortality in animal studies [3]. We present a case of West Nile encephalitis with isolated phrenic nerve involvement leading to terminal respiratory failure without any evidence of flaccid paralysis. Our case also illustrates the use of thoracic ultrasound in accurately diagnosing diaphragmatic palsy. 

## 2. Case Presentation

An 80-year-old man complaining of drowsiness was brought to the emergency room by his family. He was in his usual state of health in the previous week and started having influenza-like (flu) symptoms with on-and-off fever, body ache, lethargy, and generalized weakness. His family noticed that, for the past two days, he was sleepier than usual and remained in bed. This was mentioned as very unusual because he maintains an active lifestyle for his age. His family continued that he had recently completed a successful hunting season. He was drowsy on presentation, but oriented to all the spheres without any focal neurological deficits. A chest X-ray showed possible right lower lobe infiltrates, and he was admitted with the initial diagnosis of aspiration pneumonia. Despite appropriate antibiotics, his mental status continued to decline and developed severe encephalopathy responding only to painful stimuli. A computed tomography (CT) scan of the head did not show any acute changes. Arterial blood gas showed evidence of hypercapnic respiratory failure (Table 1).

The patient was started on bilevel positive pressure ventilation (BIPAP) for carbon dioxide narcosis. A CT chest angiogram did not show any pulmonary embolism, nor any evidence of pneumonia. Despite meticulous titration of BIPAP settings, the patient continued to have persistent hypercapnia with compensated respiratory acidosis (Table1). Based on previous serum bicarbonate levels there was no evidence of chronic respiratory failure. The CT of the chest did not show any structural defect of the pulmonary system, and the patient’s wife denied any symptoms of sleep apnea. In view of persistent encephalopathy despite compensated acidosis, further evaluation was performed. An electroencephalogram did not show any seizure activity. A lumbar puncture showed elevated protein levels (367 G/L) in the cerebrospinal fluid (CSF) and a total cell count of 13/cc with lymphocyte predominance (92%) suggesting meningoencephalitis. With supportive care, his mental condition improved, but the patient could not be weaned from BIPAP. Any interruption of BIPAP would lead to hypoxic hypercapnia respiratory failure. Meanwhile, the CSF viral panel came back as positive for West Nile virus (WNV) antibodies, both IgM and IgG. Upon further questioning, the patient’s wife revealed their recent trip to El Salvador one month ago.

As the patient was dependent of BIPAP, neurological respiratory failure due to WNV was considered. The patient did not show any evidence of acute flaccid palsy. In view of his overall condition, fluoroscopic examination for diaphragmatic movements, and detailed pulmonary functions tests to assess respiratory muscle strength were not performed. Manometry showed reduced maximum inspiratory pressure to 25 cm of water (normal: >60), and an ultrasound of the thorax showed absent movements of both diaphragms with deep inspiration (Video 1). Due to a lack of resources, electrophysiological studies of the phrenic nerve and diaphragm were not conducted. 

Based on these corroborative findings, neurological respiratory failure due to WNV encephalitis with isolated phrenic nerve involvement was diagnosed. The patient was continued on BIPAP therapy with improvement in their mental and respiratory condition, however, he succumbed to the terminal illness within two months of disease onset. Isolated phrenic nerve palsy due to WNV leading to respiratory failure is rare, and has only been reported in two previous cases in the literature [1,2].

## 3. Discussion

West Nile virus (WNV) is a single-stranded RNA virus. It belongs to the family *Flaviviradae*, which includes Japanese encephalitis virus, St. Louis encephalitis virus, Rocio virus, along with Murray valley encephalitis virus [3]. WNV is maintained in nature by enzootic transmission between birds acting as hosts and mosquitoes acting as vectors. Humans and horses are bitten by infected mosquitoes, and present as epidemic infections [3].

Most patients with WNV infections are asymptomatic [4,5]. Around 20% of infected patients develop a flu-like illness called West Nile Fever characterized by high fever, myalgia, headache, and fatigue arthralgia [4,5]. In less than 1% of patients, the WNV can infect the nervous system leading to WNND. Elderly and immunocompromised patients are more prone to neurological involvement. Although rare, the case fatality rate of WNND is approximately 10% [4]. 

WNV meningitis presents with fever, headache, and classic meningeal signs [6]. This form has a more favorable outcome compared to other forms of WNND. WNV encephalitis has a varied presentation from a mild self-limiting confessional state to severe coma and death [6]. Unlike other virus-related encephalitis, WNV has a particular predilection for extrapyramidal structures and frequently involves the brainstem, deep grey matter, and the cerebellum. Hence, the presence of extrapyramidal syndrome might help in accurate diagnosis [5]. Bilateral upper extremity and facial myoclonus is also typical of WNND [5]. Acute flaccid paralysis presents with sudden onset flaccid weakness of extremities. Direct infection of anterior horn cells by WNV results in anterior myelitis [5]. The most severe form of this disease leads to dense paraplegia with involvement of intercostal muscles and diaphragm leading to respiratory failure. In animal studies, the involvement of respiratory failure correlates with mortality [7].

West Nile Virus can also involve the peripheral nervous system leading to demyelinating neuropathies, motor axonopathy, axonal polyneuropathy, and brachial plexopathies. Involvement of the phrenic nerve leading to diaphragmatic palsy is rare [5]. Our patient initially presented with features of encephalitis without any evidence of extremity weakness. Although a formal sleep study could not be performed, interrogation of the bilevel positive airway pressure (BIPAP) machine did not show any evidence of central or peripheral sleep apnea and, despite improvement in mental status, the patient continued to show evidence of diaphragmatic palsy suggesting against central nervous system involvement in respiratory failure. Additionally, thoracic ultrasound showed absent diaphragmic excursion with inspiration, suggesting diaphragmatic weakness. 

## 4. Conclusions

West Nile virus infection can cause respiratory failure due to acute flaccid paralysis of respiratory muscles. Physicians should be wary of the rare possibility of isolated phrenic nerve involvement leading to respiratory failure, as indicated in our patient.

## Figures and Tables

**Table 1:** Serial arterial blood gas report showing persistence of hypercapnia with development of compensated respiratory acidosis.

	PH (7.35–7.45)	PCO_2_ (38–42)	PO_2_ (80–100)	HCO_3_ (18–24)	O_2_ SAT (95–98)	FIO_2_
Day 1	7.29	66	66.4	30.7	91.2	28
Day 2	7.30	64	80.3	30	95	28
Day 3	7.38	69.3	77.4	40.2	92	36
Day 10	7.35	65	103	40.7	98	28
Day 20	7.38	69	63.8	36.3	91	36
Day 30	7.32	82	74	39.7	92	28

PH = potential of hydrogen; PCO_2_ = Partial pressure of carbon dioxide; PO_2_ = Partial pressure of oxygen; HCO_3_ = Bicarbonate; O_2_ Sat = Saturation of oxygen; FIO_2_ = Fraction of inspired oxygen.
